# Pre-dialysis medical social worker support and survival in patients with kidney failure: impact on unplanned dialysis, hospitalization, and prognosis

**DOI:** 10.1080/0886022X.2025.2578417

**Published:** 2025-11-09

**Authors:** Mineaki Kitamura, Hiroshi Yamashita, Haruka Fukuda, Takuma Ishii, Emiko Otsuka, Kenta Torigoe, Takahiro Takazono, Noriho Sakamoto, Hiroshi Mukae, Tomoya Nishino

**Affiliations:** ^a^ Department of Nephrology, Nagasaki University Graduate School of Biomedical Sciences, Nagasaki, Japan; ^b^ Department of Nephrology, Nagasaki Harbor Medical Center, Nagasaki, Japan; ^c^ Department of Respiratory Medicine, Nagasaki University Graduate School of Biomedical Sciences, Nagasaki, Japan

**Keywords:** Medical social workers, social determinants of health, multidisciplinary care, unplanned dialysis initiation, hemodialysis, end-stage kidney disease

## Abstract

The role of medical social workers (MSWs) in managing patients with end-stage kidney disease (ESKD) undergoing hemodialysis remains unknown. This study evaluated the prognostic impact of pre-dialysis MSW support in patients undergoing renal replacement therapy. This retrospective analysis included 257 patients who started hemodialysis at the Nagasaki Harbor Medical Center between 2016 and 2023. Patients were divided into MSW (+) and MSW (−) groups based on whether they received pre-dialysis MSW support. Outcomes were assessed, including unplanned dialysis initiation, length of hospital stay, and post-hemodialysis survival. The MSW (+) group showed a lower rate of unplanned dialysis initiation, shorter hospital stays and improved post-hemodialysis prognosis compared with the MSW (−) group. In the multivariable Cox regression analysis, pre-dialysis MSW support (hazard ratio 0.58, 95% confidence interval 0.34–0.98, *p* = 0.04) was associated with lower patient mortality in addition to age, male sex, serum albumin levels 1 month before hemodialysis initiation, and serum creatinine levels 1 month before hemodialysis initiation. Stratified analyses showed a marked positive impact of MSW support in patients aged ≥70 years, male sex, living with family, and malnutrition. MSW support facilitated smoother transitions to maintenance hemodialysis centers through pre-settlement dialysis centers, alleviated patient anxiety, and ensured transportation arrangements. This study highlights the critical role of MSWs in improving clinical outcomes for patients with ESKD who are at higher social risks. Future multicenter studies are warranted to validate these findings and enhance their generalizability.

## GRAPHIC_ABSTRACT

**Figure:**
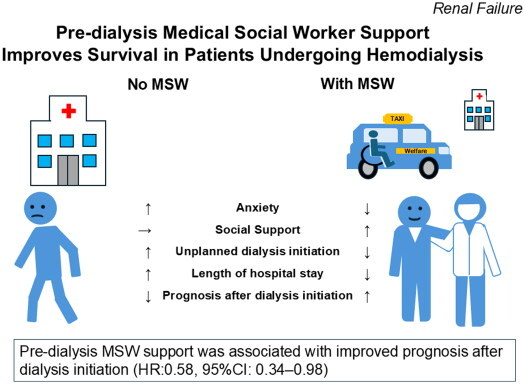

## Introduction

An increasing number of older patients worldwide are starting hemodialysis [1], a trend especially notable in developed countries, such as Japan [2]. Several problems are associated with the initiation of renal replacement therapy in older patients [3,4]. Older patients tend to have various complications, including ischemic heart disease and cerebrovascular diseases [5]. Additionally, these patients are frail and sarcopenic, which may hinder regular hospital visits for dialysis [6].

Shared decision-making (SDM) is widely recognized as fundamental for patients with end-stage kidney disease (ESKD) [3,4]. SDM enables patients with ESKD to undergo renal replacement therapy with full understanding [7]. With good communication between healthcare workers and patients, unnecessary hospitalization may be avoided [8]. Although the choice of renal replacement therapy differs by country and region, in Japan, hemodialysis is commonly preferred [2]. However, older patients often have difficulties visiting hospitals for dialysis [4]. Choosing a dialysis facility and arranging transportation facilities are often relevant concerns [9].

As patients who initiate hemodialysis become older, healthcare workers often face challenges associated with patients’ social backgrounds and healthcare problems. Medical social workers (MSWs) perform several tasks to help patients who initiate hemodialysis [10,11]. These include psychological support, advice on medical expenses, assistance with applying for public assistance, selection of hemodialysis centers, and regular hospital visits [10,11].

Our facility recommends educational hospitalization and SDM for patients with chronic kidney disease. If patients select hemodialysis as a modality for renal replacement therapy, we attempt to prepare an arteriovenous fistula in advance [12]. We also recommend that patients with ESKD meet the MSWs before initiating renal replacement therapy. If patients agree with our clinical policy, MSWs meet them and their families before initiating dialysis and make arrangements to ensure a smooth transition to maintenance hemodialysis centers. MSWs ensure the safety and regular maintenance of hemodialysis center visits.

Limited studies have evaluated the impact of MSWs on patients with ESKD. We hypothesized that pre-dialysis MSW support would improve patient prognosis. In this study, we aimed to evaluate its prognostic impact on patients initiating hemodialysis. We aimed to provide evidence for the critical role of MSWs in improving clinical and logistic outcomes for patients with ESKD starting hemodialysis.

## Materials and methods

### Patients

We included patients who initiated hemodialysis at the Nagasaki Harbor Medical Center between 2016 and 2023. Patients who started dialysis within 30 days of their first consultation with a nephrologist were excluded because 1 month would be needed in preparation for maintenance hemodialysis initiation [13]. Generally, patients approaching renal replacement therapy initiation visit doctors at least once per month at our facility. Moreover, we have a policy of having the patient, their family, and the MSW meet before starting dialysis. However, some patients are reluctant to meet MSWs because the patients wish to postpone hemodialysis initiation or disagree with initiating renal replacement therapy.

Pre-dialysis support of MSWs was confirmed using electronic medical records. The patients were divided into two groups based on whether they received pre-dialysis MSW support. Unplanned dialysis initiation was defined as the initiation of hemodialysis upon unexpected admission to our hospital. Patients who regularly visited our department but initiated hemodialysis unexpectedly at other facilities were not included because their dialysis initiation was not recorded in our database.

### Data collection

Patient data were collected at the time of the first referral to our institution and initiation of hemodialysis. Comorbidities, history of smoking, prescriptions, and medical history were documented at the time of hemodialysis initiation. Laboratory data were collected both 1 month before and at the time of hemodialysis initiation. Nutrition status was evaluated using the geriatric nutritional risk index (GNRI, cutoff: 91) as described previously [14]. Additionally, the general conditions at the time of hemodialysis initiation, such as infection, uremia, congestive heart failure, activities of daily living (ADLs), and cognitive function, were extracted from electronic medical records. ADLs and cognitive function were evaluated using “the Degree of Independence in Daily Living for the Elderly with Disabilities” and “the Degree of Independence in Daily Living for the Elderly with Dementia” issued by the Ministry of Health, Japan [15,16]. Dependent ADLs were defined as A1 and above [17], and dementia was defined as IIa and above [18]. The number of cohabiting family members, the type of insurance, and the enrollment status for the “Long-term Care Insurance System” [19] were obtained from the medical records. The “Long-term Care Insurance System” is a mandatory program providing benefits regarding older persons’ long-term care needs. Distances from the patients’ homes to our facility and maintenance hemodialysis facilities were calculated using Google Maps [20]. The patients’ 5-year prognosis following hemodialysis initiation was monitored in collaboration with maintenance dialysis centers in Nagasaki City until February 2024.

### Statistical analysis

The results are presented as percentages and counts for categorical variables and as mean ± standard deviation or median (interquartile range) for continuous variables with non-normal distributions. The chi-squared test was used to assess the association between categorical variables, whereas the Wilcoxon rank-sum test was used to assess continuous variables. A multivariable logistic regression analysis for pre-dialysis support was conducted using relevant parameters measured 1 month before hemodialysis initiation. Five-year survival was assessed using the log-rank test. Univariate and multivariate Cox regression analyses were performed for the prognostic analysis. Model 1 included age, sex, history of diabetes, history of ischemic heart disease, serum albumin level measured 1 month before hemodialysis initiation, and serum creatinine level measured 1 month before hemodialysis initiation. Model 2 included the parameters of Model 1 and pre-dialysis MSW support. Model 3 included parameters that were statistically significant in univariable Cox regression models except for body mass index (BMI) (*p* < 0.05). Using the parameters in Model 2, stratified analyses were conducted to assess the impact of the hemodialysis support of MSWs based on patients’ age (≥70 years or not), sex, BMI (≥22 or not), histories of ischemic heart disease and stroke (yes or no), serum albumin levels (≥3.5 or not), living alone (yes or no), distance from home to our facility (divided by median), and GNRI (>91 or not). Statistical significance was set at two-tailed *p* < 0.05. All statistical analyses were performed using the JMP Pro software (version 17.0; SAS Institute, Cary, NC, USA).

### Ethics

The study was approved by the Nagasaki Harbor Medical Center Institutional Review Board (approval number: R05-30) and adhered to the ethical standards of the 1964 Declaration of Helsinki and its subsequent amendments. The requirement for informed consent was waived because of the retrospective design of the study and use of anonymized data.

## Results

Of the 305 patients who initiated renal replacement therapy between 2016 and 2023, 15 underwent peritoneal dialysis, and 33 initiated hemodialysis within 30 days after the first consultation or first visit to a nephrologist, and they were excluded from this study. A total of 257 patients were included in this study, of whom 132 and/or their families received MSW support before initiating hemodialysis. The included patients were divided into MSW (+) and MSW (−) groups.

Table 1 shows demographic and laboratory data for 1 month before hemodialysis initiation. As the mean age of patients was over 70 years old, the number of patients who received employees’ health insurance (i.e. working population) was low in the MSW (+) (15.2%) and MSW (−) (10.4%) groups. As our facility is located in Nagasaki City, approximately 20% of the patients were atomic bomb survivors. The median distance from patients’ homes to our facility was 5.9 (3.5–10.4) km. There were no significant differences between the two groups except for serum creatinine levels. The history of hospitalization for patient education that included SDM for renal replacement therapy did not differ between the groups, either. In the univariable and multivariable logistic regression analyses for the MSW (+) group, only the existence of vascular access was associated with pre-dialysis MSW support (Supplementary Table 1).

**Table 1.** Baseline characteristics of patients with and without pre-dialysis medical social worker support.

	Total (*n* = 257)	MSW (+) (*n* = 132)	MSW (−) (*n* = 125)	*P* value
Age (years)	72.2 ± 11.3	72.0 ± 11.0	72.5 ± 11.7	0.61
Male (%)	164 (63.8)	82 (62.1)	82 (65.6)	0.56
BMI (kg/m^2^) 1 month before HD initiation	23.7 ± 4.6	23.9 ± 4.5	23.4 ± 4.7	0.26
Causes of ESKD (%)				0.86
Diabetic kidney disease	118 (45.9)	61 (46.2)	57 (45.6)	
Nephrosclerosis	72 (28.0)	39 (29.6)	33 (26.4)	
Glomerulonephritis	45 (17.6)	23 (17.4)	22 (17.6)	
ADPKD	3 (1.6)	1 (0.8)	2 (1.6)	
Others	19 (7.4)	8 (6.1)	11 (8.8)	
Diabetes (%)	126 (49.0)	66 (50.0)	60 (48.0)	0.75
Hypertension (%)	243 (95.7)	127 (96.2)	121 (96.8)	0.80
History of ischemic heart disease (%)	44 (17.1)	19 (14.4)	25 (20.0)	0.23
History of ischemic stroke (%)	23 (9.0)	11 (8.3)	12 (9.6)	0.72
History of cerebral hemorrhage (%)	5 (2.0)	2 (1.5)	3 (2.4)	0.61
History of smoking (%)				0.72
Never smoked	114 (44.5)	57 (43.5)	57 (45.6)	
Past smoker	79 (30.9)	41 (31.3)	38 (30.4)	
Current smoker	53 (20.7)	26 (19.9)	27 (21.6)	
Unknown	10 (3.9)	7 (5.4)	3 (2.4)	
Number of cohabiting family members	1.3 ± 1.2	1.3 ± 1.3	1.2 ± 1.0	0.88
Living alone (yes) (%)	66 (25.7)	38 (28.8)	28 (22.4)	0.24
Insurance type (%)				0.85
(1) National Health Insurance	78 (30.4)	39 (29.6)	39 (31.2)	
(2) Employee’s health insurance	33 (12.8)	20 (15.2)	13 (10.4)	
(3) Medical care system for elderly in the latter stage of life	73 (28.4)	36 (27.3)	37 (29.6)	
(4) Public assistance	22 (8.6)	11 (8.3)	11 (8.8)	
(5) Atomic bomb survivors	51 (19.8)	26 (19.7)	25 (20.0)	
Distance to our facility (km)	5.9 (3.5–10.4)	6.0 (3.5–10.3)	5.7 (3.4–10.8)	0.75
History of hospitalization for patient education	103 (40.1)	56 (42.4)	47 (37.6)	0.43
**Laboratory data 1 month before HD initiation**				
Hemoglobin (g/dL)	9.7 ± 1.4	9.6 ± 1.4	9.7 ± 1.4	0.20
Creatinine (mg/dL)	7.7 ± 2.9	7.9 ± 2.4	7.5 ± 3.4	0.03
Blood urea nitrogen (mg/dL)	83.2 ± 25.4	84.5 ± 21.5	81.8 ± 28.9	0.24
Albumin (g/dL)	3.4 ± 0.7	3.4 ± 0.7	3.4 ± 0.6	0.80
Potassium (mEq/L)	4.5 ± 0.7	4.6 ± 0.7	4.5 ± 0.7	0.60
Corrected calcium (mg/dL)	8.8 ± 0.9	8.7 ± 0.8	8.8 ± 1.0	0.66
Phosphorus (mg/dL)	5.8 ± 1.7	5.8 ± 1.5	5.8 ± 1.9	0.46
C-reactive protein (mg/dL)	0.19 (0.05–0.41)	0.20 (0.06–0.42)	0.15 (0.04–0.42)	0.27

BMI, body mass index; HD, hemodialysis; ESKD, end-stage kidney disease; ADPKD, autosomal dominant polycystic kidney disease.

Wilcoxon ranked sum test and chi-squared test were used. *p* < 0.05 was considered statistically significant.

In contrast, the clinical status during hemodialysis initiation differed between the two groups (Table 2). The rate of unplanned dialysis initiation was lower, and the length of hospital stay was shorter in the MSW (+) group versus the MSW (−) group. Although the proportion of cognitive impairment was higher in MSW (−) than in MSW (+), no significant difference was observed. However, ADLs at the time of dialysis initiation were worse in the MSW (−) group (*p* = 0.04) (Supplementary Figures 1 and 2). Additionally, the proportion of new applications for the Long Term Care Insurance System was higher in the MSW (−) group (*p* = 0.008) than in the MSW (+) group. Following a medical collaboration plan at the time of dialysis initiation, briefly, the required MSW roles are (1) reducing anxiety, (2) home medical assistance, (3) aiding rehabilitation, (4) reducing the burden of caregivers, (5) reducing medical expense, including new application for the Long-term Care Insurance System, (6) collaborating with regional facilities, and (7) support of transfer (Supplementary Table 2). Compared with those in the MSW group (22.9%), more patients (58.3%) in the MSW (-) group needed support at discharge from our hospital. Additionally, more than half of patients in the MSW (-) group were considered to be supported by the Long Term Care Insurance System. The enrollment status and requirements of the Long Term Care Insurance System are shown in Supplementary Table 3.

**Table 2.** Patient conditions and outcomes at the hemodialysis initiation with and without pre-dialysis medical social worker support.

	Total (*n* = 257)	MSW (+) (*n* = 132)	MSW (−) (*n* = 125)	P value
Duration from first referral to HD initiation (days)	480 (189–971)	504 (201–933)	466 (182–1040)	0.87
Unplanned dialysis initiation (yes) (%)	116 (45.1)	29 (22.0)	87 (69.6)	<0.001
CVC insertion (yes) (%)	35 (13.6)	7 (5.3)	28 (22.4)	<0.001
Any infections (yes) (%)	41 (16.0)	9 (6.8)	32 (25.6)	<0.001
Pneumonia (yes) (%)	29 (11.3)	6 (4.6)	23 (18.4)	0.001
Congestive heart failure (yes) (%)	97 (37.7)	32 (24.2)	65 (52.0)	<0.001
ADLs (dependent) (≥ A1) (%)	75 (29.2)	31 (23.5)	44 (35.2)	0.04
Cognitive impairment (≥ IIa) (%)	42 (16.3)	19 (14.4)	23 (18.4)	0.39
New application for “Long-term Care Insurance System” (yes) (%)	38 (14.8)	12 (9.1)	26 (20.8)	0.008
Length of hospital stay (days)	13 (7–28)	8 (5–21)	19 (10–35)	<0.001
Distance to maintenance facilities (km)	4.9 (2.7–9.0)	4.8 (2.7–8.3)	5.3 (2.8–9.4)	0.36
**Laboratory data at the HD initiation**				
Creatinine (mg/dL)	8.4 ± 3.2	8.2 ± 2.5	8.7 ± 3.9	0.83
Blood urea nitrogen (mg/dL)	90.8 ± 27.6	86.9 ± 22.5	94.9 ± 31.7	0.11
Albumin (g/dL)	3.1 ± 0.6	3.2 ± 0.6	3.1 ± 0.6	0.04
Potassium (mEq/L)	4.4 ± 0.8	4.3 ± 0.7	4.4 ± 0.9	0.36
Corrected calcium (mg/dL)	8.7 ± 0.9	8.7 ± 0.8	8.7 ± 0.9	0.61
Phosphorus (mg/dL)	6.2 ± 2.1	5.9 ± 1.8	6.4 ± 2.4	0.38
intact-parathyroid hormone (pg/mL)	235 (156–362)	267 (171–379)	205 (147–324)	0.04
C-reactive protein (mg/dL)	0.21 (0.04–1.21)	0.10 (0.02–0.40)	0.46 (0.10–2.69)	<0.001

HD, hemodialysis; CVC, central venous catheter; ADLs, activities of daily living.

**Figure 1.**
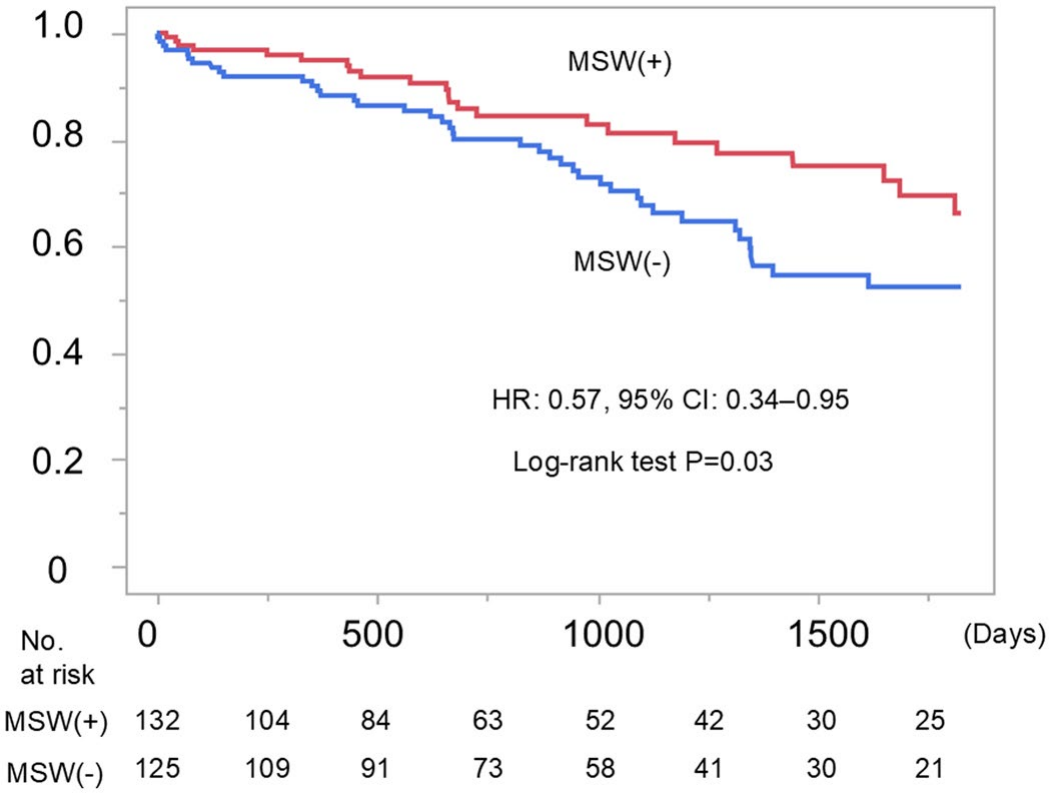
Survival analysis. MSW, medical social worker; HR, hazard ratio; CI, confidence interval. The MSW (+) group showed significantly improved survival compared with the MSW (-) group.

The median observational period was 801 (interquartile range, 379–1440) days after hemodialysis initiation. Sixty-four patients died during the observational period. The most common cause of death was infections (*n* = 21), followed by cardiovascular diseases (*n* = 13). There was a significant difference in the post-hemodialysis initiation prognosis between the MSW (+) and MSW (−) groups (Figure 1). As the patients who had infections were significantly higher in the MSW (−) group, we analyzed patient survival curves according to its presence (infections (+)) or absence (infections (−)) and found that patients with infections showed poor outcomes (Supplementary Figure 3). The results of the Cox regression analysis are presented in Table 3. Multivariable Cox regression analysis (Model 1) showed that age, male sex, serum albumin levels, and serum creatinine levels were risk factors for death. Additionally, Model 2 showed that pre-dialysis MSW support decreased the risk of death.

**Table 3.** Univariable and multivariable Cox regression analysis for prognosis after the initiation of hemodialysis.

	Univariable			Model 1			Model 2			Model 3		
	HR	95% CI	P value	HR	95% CI	P value	HR	95% CI	P value	HR	95% CI	P value
Age	1.06	1.04–1.09	<0.001	1.04	1.01–1.07	<0.001	1.04	1.01–1.07	<0.001	1.04	1.01–1.07	<0.001
Male sex	1.54	0.89–2.66	0.12	1.93	1.10–3.43	0.02	1.84	1.04–3.28	0.04			
BMI 1 month before HD initiation	0.92	0.86–0.98	0.01									
Diabetes mellitus (yes)	0.98	0.60–1.60	0.92	0.90	0.54–1.51	0.70	0.92	0.75–1.54	0.75			
History of IHD (yes)	1.85	1.07–3.20	0.03	1.57	0.89–2.79	0.12	1.52	0.86–2.69	0.15	1.46	0.81–2.62	0.21
Hemoglobin 1 month before HD initiation	1.01	0.84–1.21	0.96									
Serum albumin 1 month before HD initiation	0.37	0.24–0.59	<0.001	0.52	0.33–0.82	0.006	0.49	0.31–0.77	0.002	0.44	0.27–0.71	<0.001
Serum creatinine 1 month before HD initiation	0.81	0.72–0.90	<0.001	0.87	0.77–0.99	0.03	0.89	0.79–1.01	0.07	0.91	0.78–1.06	0.22
Serum corrected calcium 1 month before HD initiation	1.31	1.01–1.70	0.04							0.87	0.61–1.24	0.44
Serum phosphorus 1 month before HD initiation	0.82	0.69–0.96	0.01							0.99	0.79–1.23	0.92
Living Alone (yes)	0.99	0.58–1.76	0.97									
Pre-dialysis MSW support (yes)	0.57	0.34–0.95	0.03				0.58	0.34–0.98	0.04	0.54	0.32–0.91	0.02

HR, hazard ratio; CI, confidence interval; BMI, body mass index; HD, hemodialysis; IHD, ischemic heart disease; MSW, medical social worker.

Even Model 3, using parameters that had statistical significance in the univariable Cox regression model, showed that pre-dialysis MSW support decreased the risk of death.

**Figure 2.**
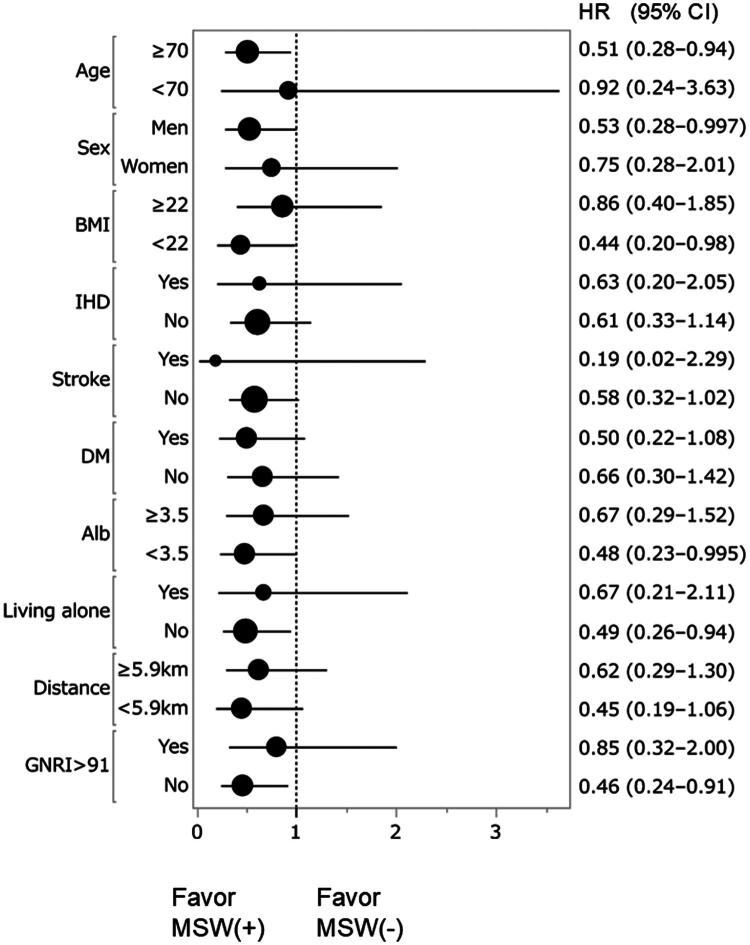
Stratified Cox regression analysis for patient survival. BMI, body mass index; IHD, ischemic heart disease; DM, diabetes mellitus; Alb, albumin; GNRI, geriatric nutritional risk index HR, hazard ratio; CI, confidence interval. Error bars indicate 95% confidence intervals. In the stratified multivariable Cox regression analysis, pre-dialysis medical social worker intervention significantly improved prognosis in patients aged ≥70 years, male sex, BMI< 22 kg/m^2^, Alb< 3.5 g/dL, not living alone, and GNRI < 91.

Stratified Cox regression analysis showed that pre-dialysis MSW support was more favorable in patients aged ≥70 years, men, patients not living alone, and those with BMI <22 kg/m^2^, albumin <3.5 g/dL, and GNRI <91 (Figure 2).

## Discussion

**Figure 3.**
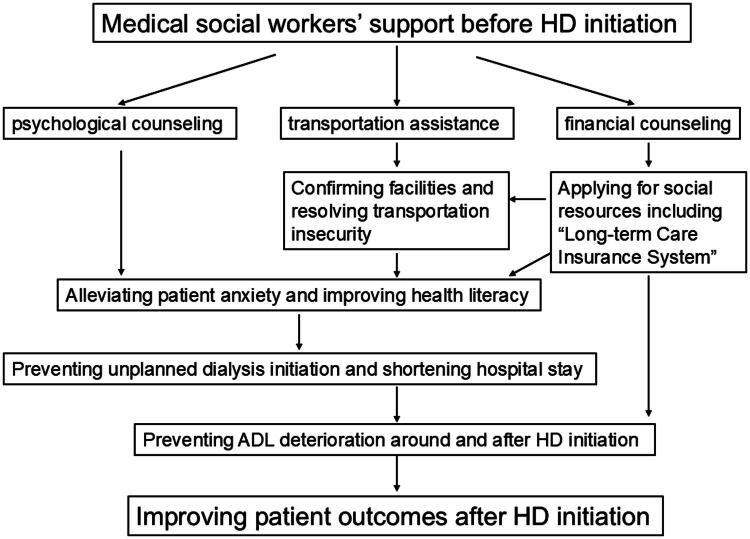
Possible positive effect of medical social workers on patients initiating hemodialysis. The impact of medical social workers on patient survival is likely to be multifactorial; however, the key contributing factors are illustrated in this scheme. One of the most important factors is preventing unplanned dialysis initiation.

We conducted an observational analysis of the prognostic impact of MSWs on patients initiating hemodialysis. To our knowledge, this is the first quantitative study linking pre-dialysis medical social worker support with survival in patients with kidney failure. Although almost all patient backgrounds were identical between the MSW (+) and MSW (−) groups, the patient prognostic outcome differed between the groups, suggesting the pre-dialysis MSW support had a positive impact on patient outcomes, especially in older male patients with malnutrition. We speculate that pre-dialysis MSW support has favorable effects in terms of transportation assistance, psychological counseling, and financial counseling (Figure 3).

Although MSWs play a relevant role in the dialysis field [10,11], past studies on the impact of MSWs have focused on psychological support for patients with ESKD [21]. Patients with ESKD tend to have complicated depression and psychological issues, and more than half of the patients who initiate dialysis have anxiety about starting dialysis [22]. A systematic review showed that MSWs help relieve psychological disorders, such as anxiety and depression, in patients with ESKD [21]. A previous study showed that more than half of the patients who initiated dialysis had anxiety about starting dialysis [22]. Although we could not evaluate the patients’ mental status, the unplanned dialysis initiation rate was lower in the MSW (+) group versus the MSW (-) group. This may suggest that pre-dialysis MSW support helped to decrease the anxiety of patients about their new lifestyles after starting hemodialysis. Older patients with renal impairment are asked to receive SDM, as are younger patients [23]. However, deciding on modalities before preparing for renal replacement therapy is sometimes challenging [24]. This may be because the life burden after the initiation of renal replacement therapy is not accepted by the patients and their families. According to the official medical collaboration plan issued shortly after admission to the hospital, seven major roles for MSWs are required to prepare for discharge (Supplementary Table 2). Reducing anxiety and home medical assistance are required for almost all patients for whom hemodialysis is initiated. However, as MSW (-) patients did not maintain good ADLs at the time of hemodialysis initiation, a rehabilitation plan after discharge and transportation aid to move to other facilities were required to be prepared by MSWs before discharge. The multivariable logistic regression analysis for pre-dialysis referral revealed that the presence of vascular access 1 month prior to hemodialysis initiation was associated with MSW referral, suggesting that such patients may be more likely to accept hemodialysis initiation. We could not evaluate health literacy for hemodialysis acceptance in this study, but it might have played a role in early MSW referral. Nevertheless, the history of education entrance and duration from first referral to the nephrology department were not associated with MSW referral. Accessibility to MSW may have differed according to the level of patients’ ADLs, and serum creatinine levels 1 month before hemodialysis initiation in the MSW (-) group reflect the poor ADL levels of these patients. Additionally, more patients in this group might have deteriorated ADLs at the time of hemodialysis initiation compared with MSW (+) group patients (Table 2 and Supplementary Figure 1b). In fact, these patients had to apply to the Long Term Care Insurance System after the initiation of hemodialysis. This study showed that the pre-dialysis MSW support would be more beneficial for specific patients, including patients of male sex, as well as those with older age and malnutrition. However, there were no significant differences in insurance type and distance from home to facilities between the MSW (+) and MSW (-) groups. Additionally, these factors were not associated with patient survival. We speculate that equal access to medical treatment, regardless of annual income, proved effective in Japan. Notably, stratified analyses show that pre-dialysis MSW referral would be profitable in patients not living alone. Although there might be a selection bias, family support does not appear to function as expected. Nevertheless, pre-dialysis MSW support will help initiate hemodialysis smoothly and might support the patients after hemodialysis initiation.

MSW support is expected to shorten the length of hospital stay for patients with ESKD [25]. In addition to treating comorbidities, social settlements sometimes delay patient discharge [25]. Coordinating maintenance hemodialysis centers after hemodialysis initiation is sometimes challenging [26]. Previous studies have shown that the lack of care coordination between hospitals and outpatient dialysis facilities contributes to health deterioration during and after hospitalization [27]. As medical resources in emergency hospitals are limited, good cooperation between dialysis centers and emergency hospitals is desired [26]. Some of these issues can be resolved if patients receive pre-dialysis MSW support. Additionally, patients who received pre-dialysis MSW support could be discharged from our hospital earlier because negotiations between our facility and dialysis centers shortened the hospital stay. The length of hospital stay is associated with deteriorating ADLs in patients undergoing hemodialysis [28], which may be associated with poor outcomes. In particular, older patients often experience a decline in ADLs when considering entry into nursing homes during hemodialysis initiation [29]. As shown in Supplementary Table 2, establishing support with regional facilities and transportation are among the crucial roles of MSWs in initiating hemodialysis. In this study, pre-dialysis MSW support shortened the length of hospital stay.

Hemodialysis requires three treatment sessions per week; therefore, it is challenging for patients to confirm dialysis transportation. Dialysis transportation cost is a primary concern in North America [30,31]. A recent study from the US showed that transportation insecurity had a negative impact on patient survival [32]. Similarly, dialysis transportation is a major mobility issue because patients undergoing hemodialysis are less active and cannot move independently. Some Japanese patients undergoing hemodialysis rely on private transfer services supported by facilities [33] or welfare vehicles [34]. The MSWs at our facility are responsible for confirming transportation arrangements to ensure that patients starting hemodialysis do not face difficulties after discharge (Supplementary Table 2). If a patient’s ADLs deteriorate during hemodialysis initiation, our MSWs provide support by supplying necessary social services, such as rehabilitation options after discharge.

This study has some critical limitations. Although we excluded patients who initiated hemodialysis within 30 days after the first referral or consultation with nephrologists, pre-dialysis MSW support may have been limited to patients in good general condition. In other words, there might be differences in ADLs 1 month before hemodialysis initiation. The serum creatinine levels 1 month before hemodialysis initiation in the MSW (−) group were lower than in the MSW (+) group. Even though ADLs deteriorated within 1 month, there was a significant difference in ADLs between the MSW (+) and MSW (−) groups at the time of hemodialysis initiation. Moreover, the proportion of new applications to the Long Term Care Insurance System was higher in the MSW (-) group. As this study was conducted retrospectively, data on patient socioeconomic status and regional effects were partially collected or evaluated. Unmeasured confounding factors might have existed and affected the results. Most dialysis centers in Nagasaki City offer shuttle bus services for patients to visit their facilities. However, not all patients could use this service because of the region and location of the dialysis facilities. Owing to the actual equality in accessibility to medical treatment in Japan, the type of insurance had no impact on patient outcome; however, this finding is not generalizable outside of Japan. Particularly, patients aged ≥75 years are supposed to pay minimal medical costs under the Japanese insurance system. Further, as this study was conducted in Nagasaki City, atomic bomb survivors were included, which may have affected the results. Also, the geographic features of Nagasaki City may have influenced the results. Owing to unmeasured confounding factors, the generalizability of the findings of this study is limited. Multicenter prospective studies are needed to validate these findings.

In conclusion, this study indicated a positive effect of pre-dialysis MSW support on patients with ESKD. We should identify patients with higher social risks, such as male sex, older age, and malnutrition, and support them before hemodialysis initiation. The role of MSWs is crucial in team medical care when starting dialysis therapy.

## Supplementary Material

Supplemental Material

Supplemental Material

Supplemental Material

Supplemental Material

Supplemental Material

## Data Availability

The datasets analyzed during the current study are available from the corresponding author upon reasonable request.
