# Supplementary figures and images for: Pre-dialysis medical social worker support and survival in patients with kidney failure: impact on unplanned dialysis, hospitalization, and prognosis
Source: Ren Fail. 2025 Nov 9;47(1):2578417. doi: 10.1080/0886022X.2025.2578417 (PMC12604136; doi:10.1080/0886022X.2025.2578417)

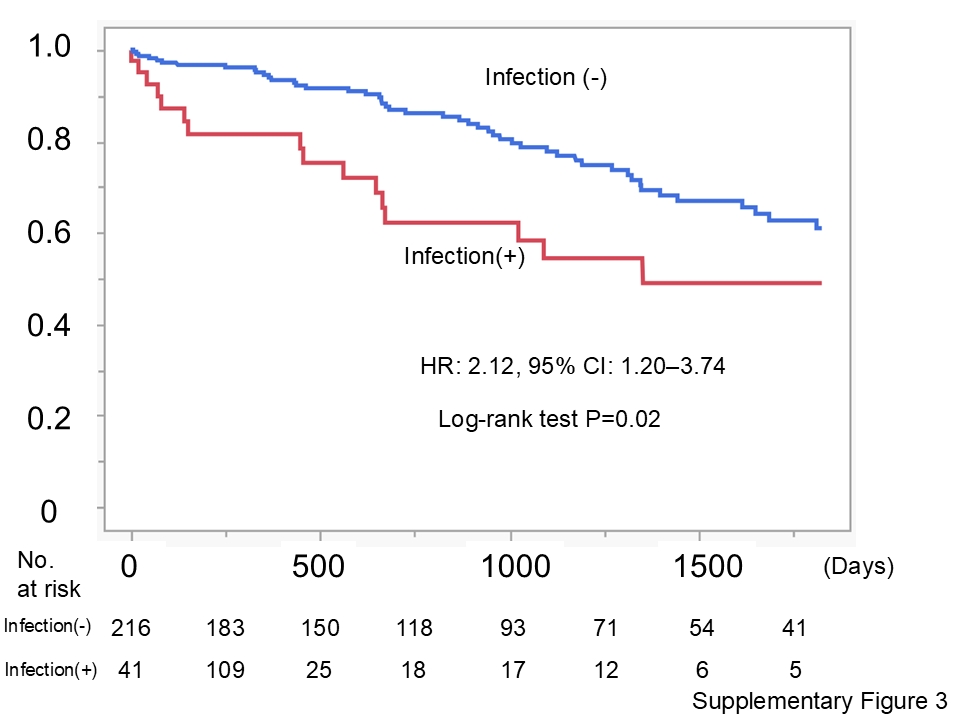

Supplement: Supplemental Material [file IRNF_A_2578417_SM3809.tif]

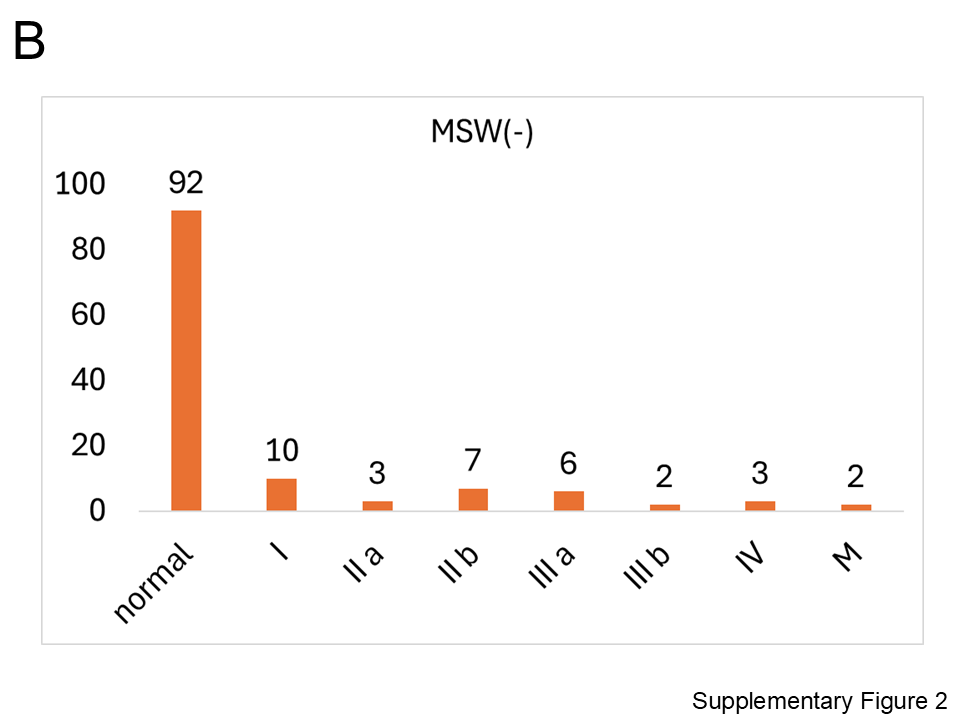

Supplement: Supplemental Material [file IRNF_A_2578417_SM3808.tif]

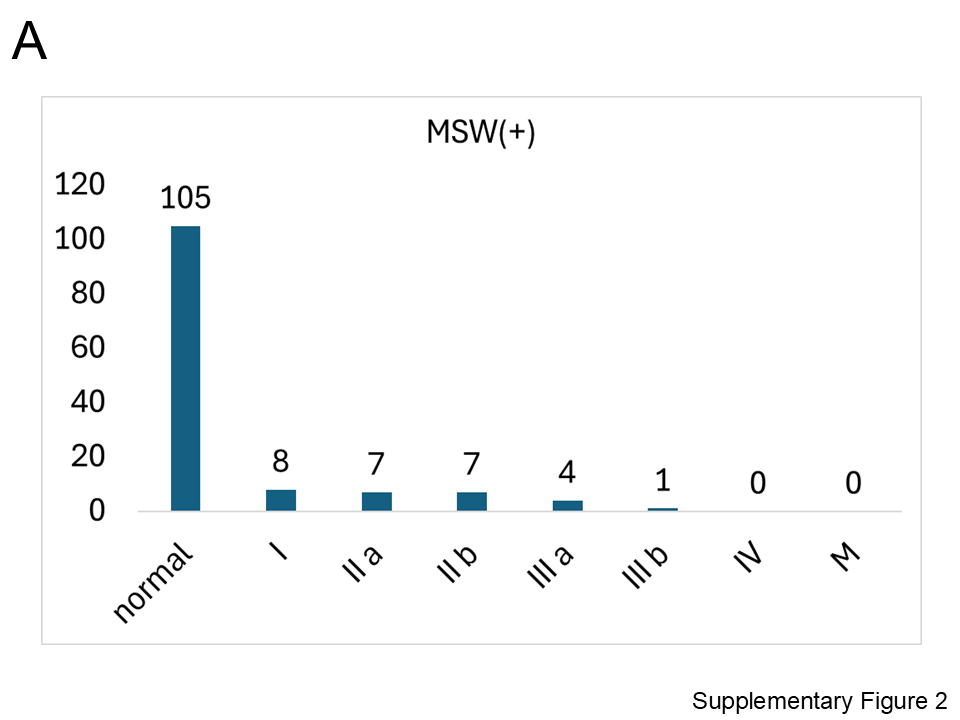

Supplement: Supplemental Material [file IRNF_A_2578417_SM3807.tif]

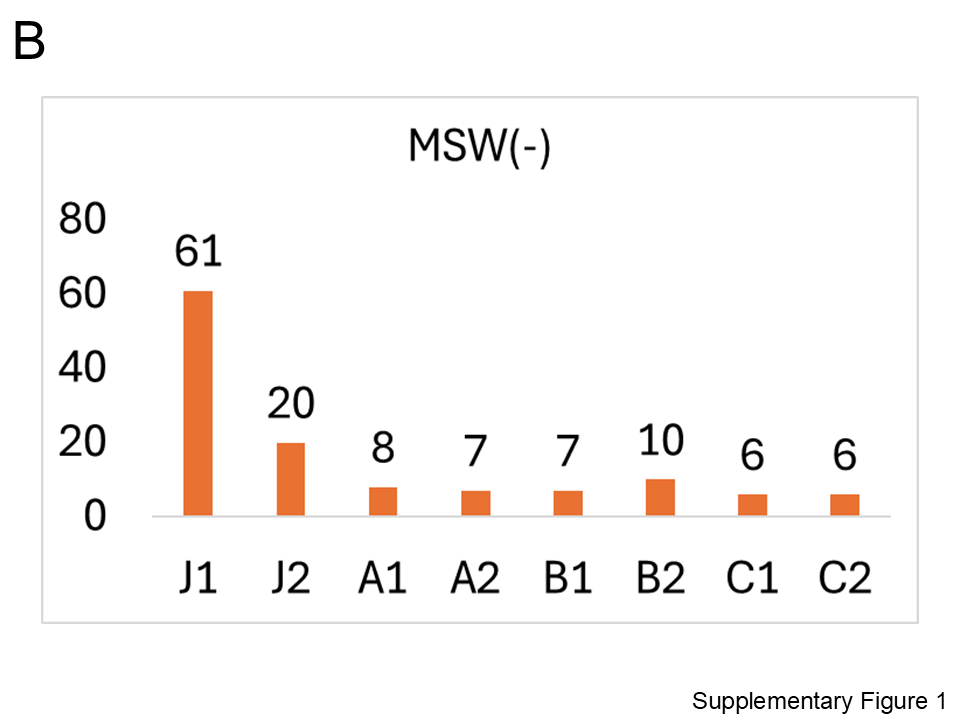

Supplement: Supplemental Material [file IRNF_A_2578417_SM3806.tif]

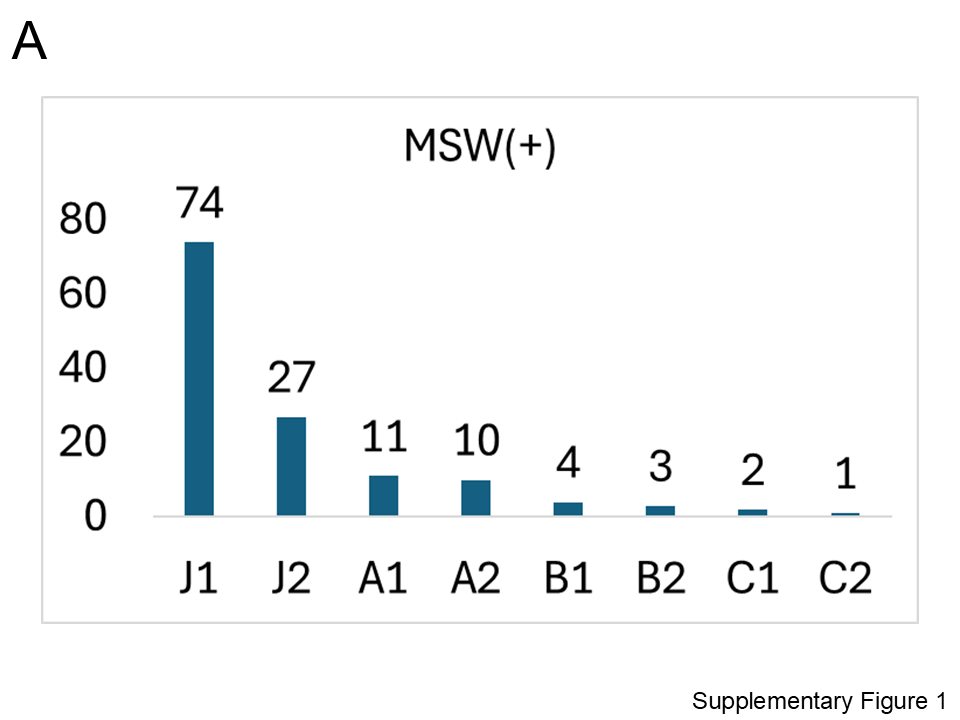

Supplement: Supplemental Material [file IRNF_A_2578417_SM3805.tif]
